# CT-derived myosteatosis as a predictor of postoperative outcomes in esophagogastric cancer surgery: a systematic review

**DOI:** 10.3389/fmed.2026.1885900

**Published:** 2026-07-21

**Authors:** Goran Rondovic, Momir Sarac, Ivan Lekovic, Nebojsa Maric, Sanja Sarac, Dusica Stamenkovic, Maja Stojanovic, Suzana Bojic

**Affiliations:** 1 Clinic for Anesthesiology and Critical Care, Military Medical Academy, Belgrade, Serbia; 2 Medical Faculty of Military Medical Academy, University of Defence, Belgrade, Serbia; 3 Clinic for Vascular and Endovascular Surgery, Military Medical Academy, Belgrade, Serbia; 4 Clinic for Chest Surgery, Military Medical Academy, Belgrade, Serbia; 5 Pulmonology Clinic, Military Medical Academy, Belgrade, Serbia; 6 Faculty of Medicine, University of Belgrade, Belgrade, Serbia; 7 EuroPeriscope, ESAIC Onco-Anesthesiology Research Group (ESAIC-RG-EP), Brussels, Belgium

**Keywords:** cancer, esophagectomy, gastrectomy, muscle quality, myosteatosis, postoperative complications, surgery

## Abstract

**Introduction:**

Myosteatosis, reflecting impaired skeletal muscle quality and fatty muscle infiltration, has emerged as a potentially important marker of perioperative vulnerability in oncologic surgery. However, evidence regarding CT-derived muscle quality parameters and postoperative outcomes after esophagogastric cancer surgery remains heterogeneous.

**Methods:**

A systematic review was conducted according to PRISMA 2020 guidelines. PubMed/MEDLINE was searched for studies evaluating CT-derived muscle quality parameters and postoperative outcomes in adult patients undergoing elective surgery for esophageal or gastric malignancies. Data regarding CT methodology, muscle quality assessment, body composition parameters, and postoperative outcomes were extracted and narratively synthesized.

**Results:**

Twenty-two studies comprising 6,733 patients were included. Myosteatosis was consistently associated with major postoperative complications (Clavien–Dindo ≥ III), with reported effect sizes generally ranging from approximately 2 to 5 across multiple cohorts. Associations with overall morbidity, specific complications, and length of stay were more heterogeneous. In several studies, muscle quality demonstrated stronger prognostic associations than muscle quantity alone, while composite body composition measures integrating muscle quality with muscle quantity, adiposity, or functional parameters showed improved predictive performance.

**Discussion:**

CT-derived myosteatosis appears to be an important marker of major postoperative morbidity in esophagogastric oncologic surgery and may provide complementary prognostic information beyond muscle quantity alone. However, substantial methodological heterogeneity limits comparability between studies and clinical implementation. Future research should prioritize standardization of muscle quality assessment and validation of clinically accessible assessment approaches.

## 1 Introduction

Traditionally viewed as a mechanical organ responsible for locomotion, skeletal muscle is now recognized as an important regulator of metabolic homeostasis and physiological resilience (1), particularly during states of stress such as surgery (2) and critical illness (3). Previous research largely focused on the relationship between reduced muscle mass, the concept of sarcopenia, and adverse outcomes across multiple diseases, including cancer (4, 5).

More recently, attention has shifted toward muscle quality, a multidimensional construct reflecting its structural integrity and the ability to perform physiological functions such as force generation, contraction–relaxation dynamics, and metabolic and endocrine activity (6). In clinical and research settings, muscle quality is commonly assessed through two complementary domains: muscle function and muscle composition (7). Muscle composition assessment largely focuses on myosteatosis, defined as pathological lipid accumulation within skeletal muscle, which has been associated with impaired muscle performance, metabolic dysfunction, systemic inflammation, and adverse clinical outcomes (8). Myosteatosis can be evaluated using several imaging modalities, most commonly computed tomography (CT), but also magnetic resonance imaging, ultrasound, and dual-energy X-ray absorptiometry. Although MRI is generally considered the reference standard, CT remains the most widely used modality in oncologic populations because it is routinely performed for cancer staging and preoperative evaluation, enabling opportunistic assessment of muscle quality without additional imaging, cost, or radiation exposure (8).

In patients undergoing cancer surgery, accurate preoperative risk stratification is essential given the substantial physiological demands of major oncologic procedures and the frequent presence of comorbidities and cancer-related metabolic alterations. Although reduced skeletal muscle mass and sarcopenia have been widely investigated as predictors of postoperative morbidity (9), growing evidence suggests that CT-derived markers of muscle quality, particularly myosteatosis, may provide additional prognostic information beyond muscle quantity alone (10). This association has been most consistently demonstrated in colorectal, pancreatic, and hepatic cancer surgery, where myosteatosis has been linked with increased postoperative morbidity, including severe postoperative complications (11–13). However, its prognostic role in esophagogastric cancer surgery remains less clearly defined. Therefore, this systematic review summarizes current approaches to CT-derived muscle quality assessment and evaluates its association with early postoperative outcomes in patients undergoing esophagogastric cancer surgery.

## 2 Methods

This systematic review was conducted and reported in accordance with the Preferred Reporting Items for Systematic Reviews and Meta-Analysis (PRISMA) 2020 guidelines. Eligibility criteria were defined using the Population, Intervention (or Exposure), Comparison, Outcomes, and Study (PICOS) framework.

### 2.1 Search strategy

A systematic literature search of PubMed/MEDLINE, Embase, Web of Science, Scopus, and the Cochrane Library from database inception to April 15th 2026. Following peer review, the search was repeated on June 20, 2026, to ensure that no eligible studies had been missed. The search strategy combined terms related to muscle quality and myosteatosis with terms related to esophagogastric cancer and surgical treatment. Search terms included combinations of “muscle quality,” “myosteatosis,” “skeletal muscle density,” “muscle attenuation,” “muscle radiodensity,” “intramuscular adipose tissue,” “IMAC,” “IMAT,” “skeletal muscle gauge,” “gastric cancer,” “esophageal cancer,” “gastrectomy,” “esophagectomy,” “postoperative complications,” and “surgical outcomes.” Reference lists of relevant articles and review papers were additionally screened to identify further eligible studies. The complete database-specific search strategies are provided in Supplementary File 1.

### 2.2 Eligibility criteria

Studies were eligible if they included adult patients undergoing elective surgery for gastric or esophageal malignancies, evaluated preoperative CT-derived muscle quality parameters, and reported postoperative clinical outcomes, including postoperative complications, major complications, length of stay, mortality, readmission, or other perioperative outcomes. Only full-text articles published in peer-reviewed journals were included. Studies involving mixed oncologic surgical cohorts were considered eligible if patients undergoing esophagogastric surgery represented part of the study population. Studies focused exclusively on non-surgical treatment, long-term survival without perioperative outcomes, animal studies, conference abstracts, editorials, reviews, and case reports were excluded.

### 2.3 Study selection

**Figure 1:**
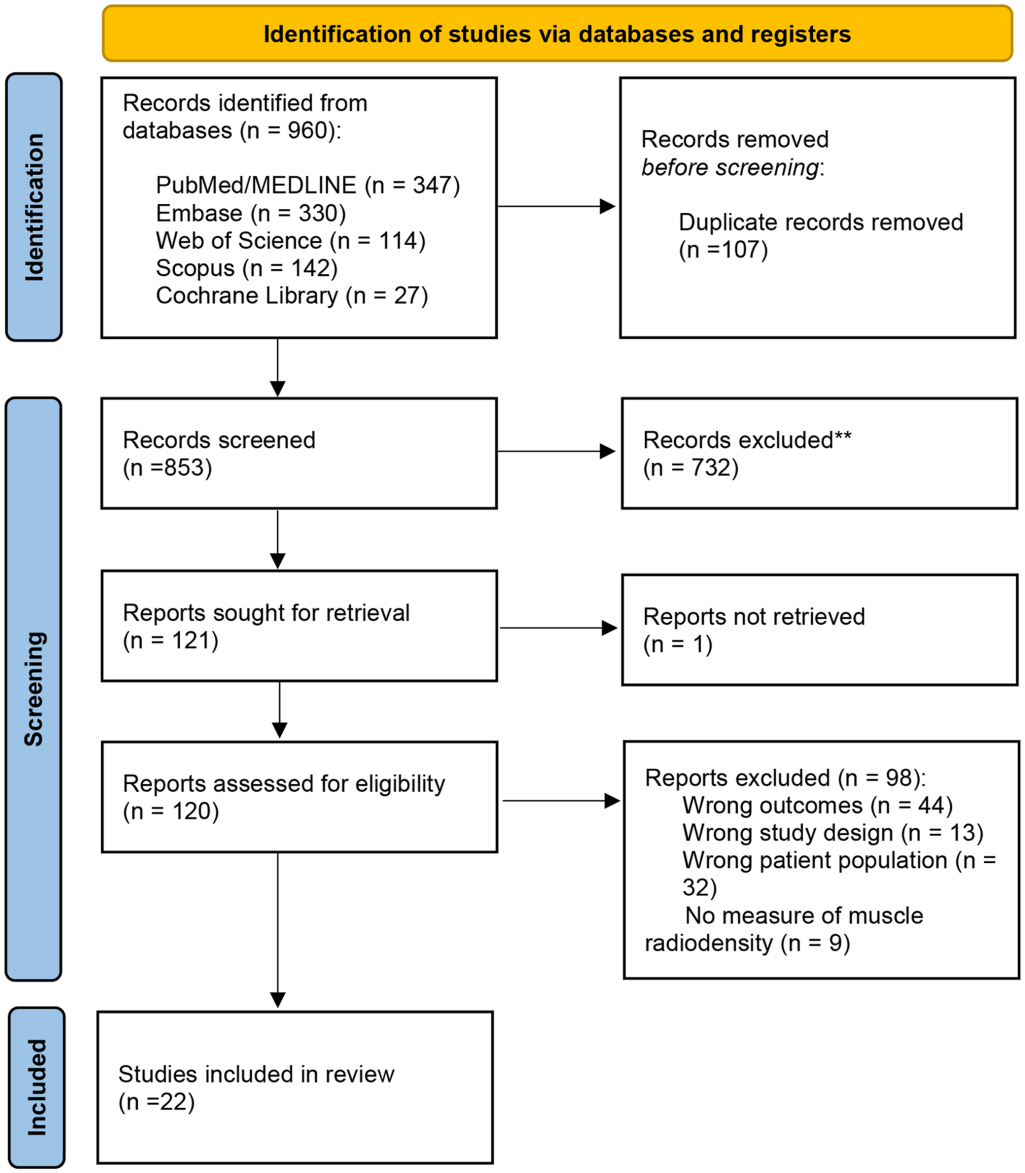
PRISMA 2020 flow diagram of study selection.

Titles and abstracts identified through the database search were screened for eligibility, followed by full-text review of potentially relevant articles. Studies meeting predefined eligibility criteria were included in the final analysis (Figure 1).

### 2.4 Data extraction

Data extraction was performed manually from all included studies. Extracted variables included study characteristics, cancer type, surgical procedure, sample size, CT acquisition protocol, anatomical level of assessment, segmentation software, analyzed muscle groups, tissue attenuation thresholds, muscle quality parameters, postoperative outcomes, and comparisons with other body composition variables. Particular attention was given to methodological aspects of muscle quality assessment, including use of contrast-enhanced versus unenhanced CT imaging, vertebral level selection, segmentation workflow, tissue attenuation thresholds, and specific muscle quality metrics.

### 2.5 Outcomes

The primary outcomes of interest were overall postoperative complications and major postoperative complications defined according to the Clavien–Dindo (CD) classification. Secondary outcomes included infectious complications, pulmonary complications, anastomotic leakage, perioperative mortality, readmission, intensive care unit admission, and length of hospital stay.

### 2.6 Data synthesis

Due to substantial methodological heterogeneity across studies regarding imaging protocols, segmentation approaches, muscle quality definitions, cut-off values, and outcome reporting, quantitative meta-analysis was not performed. Findings were therefore synthesized narratively and summarized according to imaging methodology, muscle quality measures, and postoperative outcomes.

## 3 Results

### 3.1 Study selection

The literature search and study selection process are presented in Figure 1. Twenty-two studies met the predefined eligibility criteria and were included in the final analysis.

Included studies evaluated a total of 6,733 patients undergoing surgery for esophageal or gastric malignancies. Ten studies focused on gastric cancer patients undergoing gastrectomy (14–23), while seven included exclusively esophageal cancer patients undergoing esophagectomy (24–30). One study evaluated a combined esophagogastric cohort undergoing either gastrectomy or esophagectomy (31). In addition, four mixed oncologic surgical cohorts included patients undergoing esophagogastric procedures alongside other major abdominal cancer surgeries (32–35). All included studies evaluated adult patients undergoing elective oncologic surgery. Detailed study characteristics are summarized in Tables 1, 2.

**Table 1:** Methodological approaches to CT-derived myosteatosis assessment.

Study	Cancer type (type of surgery)	Number of patients	Imaging modality	Anatomical site	Tissue segmentation software	Muscle group(s) analyzed	Tissue-specific attenuation thresholds (HU)	Muscle quality measure
Esophagogastric								
Tankel et al. (24)	Locally advanced esophageal adenocarcinoma (transthoracic en bloc esophagectomy with D2 lymphadenectomy)	105	Portal venous phase	Upper border of L3	3D Slicer	Total skeletal muscle area	Skeletal muscle: −29 to +150 HU	SMD
Guo et al. (14)	Gastric cancer (robotic radical gastrectomy)	381	Portal venous phase	L3	PACS	Total skeletal muscle area	Skeletal muscle: −29 to +150 HU; Subcutaneous adipose tissue: −190 to −30 HU; Visceral adipose tissue: −150 to −50 HU	Skeletal muscle radiodensity (SMD)
Shuto et al. (25)	Esophageal cancer (thoracic esophagectomy with lymphadenectomy)	70	NUnenhanced	L3	Fat Tissue Analyzer v3.0 (Virtual Place Fujin Raijin; Canon Medical Systems)	Bilateral psoas major muscles	Automated	Psoas muscle density (muscle attenuation, HU)
Akmercan et al. (15)	Gastric cancer (radical gastrectomy)	237	Contrast status not specified	L3	PACS (Infinitt Healthcare, Seoul, South Korea)	Total skeletal muscle area	Skeletal muscle: −29 to +150 HU	SMD
Ding et al. (16)	Gastric cancer (robotic radical gastrectomy)	381	Portal venous phase	L3	PACS	Total skeletal muscle area	Skeletal muscle: −29 to +150 HU	SMD
Zhao et al. (26)	Esophageal squamous cell carcinoma (esophagectomy)	85	Contrast status not specified	L3	Not reported	Total skeletal muscle area	Skeletal muscle: −29 to +150 HU	SMD
Zhong et al. (19)	Gastric cancer (laparoscopic radical gastrectomy)	717	Contrast status not specified	L3	SliceOmatic v5.0	Total skeletal muscle area	Skeletal muscle: −29 to +150 HU	SMRA; SMG
Park et al. (30)	Esophageal cancer (esophagectomy)	462	Contrast enhanced	L3	Not reported	Total skeletal muscle area	Skeletal muscle: −29 to +150 HU	SMD
Cameron et al. (27)	Esophageal cancer (esophagectomy)	108	Unenhanced	T4, L3	SliceOmatic v6.7	Total skeletal muscle area	Skeletal muscle: −29 to 150 HU; adipose tissue: −150 to −50 HU	MRA
Murnane et al. (31)	Esophageal and gastric cancer (radical esophagectomy or gastrectomy)	108	Portal venous phase	L3	SliceOmatic v5.0	Total skeletal muscle area	Skeletal muscle: −29 to +150 HU; Subcutaneous adipose tissue: −190 to −30 HU; Visceral adipose tissue: −150 to −50 HU; Intramuscular adipose tissue: −190 to −30 HU	SMD
Huang et al. (20)	Gastric cancer (radical gastrectomy)	597	Contrast status not specified	L3	GE ADW 4.5	Total skeletal muscle	Skeletal muscle: −29 to +150 HU; Subcutaneous fat: −190 to −30 HU; Visceral fat: −150 to −50 HU	SMD
Kemper et al. (28)	Esophageal cancer (esophagectomy)	98	Portal venous phase	L3	ImageJ	Posterior paraspinal muscles	Not reported	MRA
Matsui et al. (17)	Gastric cancer (gastrectomy)	840	Contrast status not specified	umbilicus	Ziostation	Multifidus muscles	Manual tracing with automated quantification	IMAC
Uchida et al. (18)	Gastric cancer (radical gastrectomy)	353	Contrast-enhanced	L3	SYNAPSE VINCENT	Multifidus muscles	Skeletal muscle: −29 to +150 HU	IMAC
Srpcic et al. (29)	Esophageal cancer (esophagectomy with curative intent)	139	Unenhanced	L3	ABACS	Total skeletal muscle	Skeletal muscle: −29 to +150 HU	MA
Zhuang et al. (21)	Gastric cancer (radical gastrectomy)	973 (584 after propensity score matching)	Contrast status not specified	L3	GE ADW 4.5 workstation / PACS	Total skeletal muscle area	Skeletal muscle: −29 to +150 HU	SMD
Lin et al. (22)	Gastric cancer (radical gastrectomy)	594	Contrast status not specified	L3	PACS	Total skeletal muscle area	Skeletal muscle: −29 to +150 HU	HUAC; SMG
Zhang et al. (23)	Gastric cancer (radical gastrectomy)	156	Contrast status not specified	[35, 36]	OsiriX v8.5.2	Total skeletal muscle area	Skeletal muscle: −29 to 150 HU; subcutaneous adipose tissue: −190 to −30 HU; visceral adipose tissue: −150 to −50 HU	Mean MA
Mixed cohorts								
Goda et al. (32)	Upper abdominal cancer (esophagectomy or pancreaticoduodenectomy)	46	Unenhanced	T4, L3	Aquarius iNtuition	Total skeletal muscle area	Skeletal muscle: − 29 to 150 HU; subcutaneous adipose tissue: − 190 to −30 HU; visceral adipose tissue: − 150 to −50 HU	Mean SMD (mean MA)
Cereda et al. (33)	Upper gastrointestinal cancer (pancreatoduodenectomy or total gastrectomy)	104	Unenhanced	L3	ImageJ	Total skeletal muscle area	Skeletal muscle: −29 to +150 HU; intramuscular adipose tissue: −190 to −30 HU	Skeletal MA
Gagnat et al. (34)	Digestive cancer (major elective abdominal surgery)	95	Contrast status not specified	L3	PACS, Telemis 4.7	Multifidus muscles	Skeletal muscle: − 29 to 150 HU; IMAT defined as fat infiltration into multifidus muscles of −190 to −30 HU	IMAT
Carvalho et al. (35)	Gastrointestinal cancer (elective open resection)	84	Contrast status not specified	L3	SliceOmatic v5.0	Total skeletal muscle area	Skeletal muscle: −29 to +150 HU; visceral adipose tissue: −150 to −50 HU	Skeletal muscle radiodensity (SMD)

CT, computerized tomography; SMD, Skeletal muscle density; SMRA, Skeletal muscle radiation attenuation; SMG, skeletal muscle gauge; MRA, Muscle radiation attenuation; IMAC, Intramuscular adipose tissue content; MA, Muscle attenuation; HUAC, Hounsfield unit average calculation; IMAT, Intermuscular adipose tissue.

**Table 2:** Associations of CT-derived myosteatosis with early postoperative outcomes in patients undergoing esophagogastric surgery.

Study	Cut-off values	Any complication (CD I - V)	Major complications (CD ≥ III)	Other specific complications	Length of stay	Other body composition variables tested	Main conclusion
Esophagogastric							
Tankel et al. (24)	SMD < 33.8 HU (males), < 33.6 HU (females),	Not reported	36.4% vs. 15.3%, *p* = 0.009	Not reported	Not significant	Muscle quantity: SMI	Persistent/developed myosteatosis but not sarcopenia during neoadjuvant chemotherapy was associated with increased major postoperative complications.
Guo et al. (14)	SMD < 38.5 HU (males), < 28.6 HU (females)	OR 2.49 (*p* < 0.001)	Not reported separately for SMD alone	Unplanned ICU admission: OR 2.00 (*p* = 0.004); 30-day mortality: OR 7.34 (*p* < 0.001)	SMD: not significant lSMI + SMD: OR 2.67 (95% CI: 1.71–4.79, *p* < 0.05)	Muscle quantity: SMI; Adipose: VATI, SATI, BMI	Both muscle quality and muscle quantity predicted postoperative complications, unplanned ICU admission, and 30-day mortality.
Shuto et al. (25)	Sex-specific: muscle area <5.68/2.52 cm^2^/m^2^, muscle density <92.9/80.6 HU, fat area <58.6/20.0 cm^2^/m^2^ (male/female). BC score 0–3 based on number of parameters above cut-off.	Unfavorable vs. favorable BC: 92% vs 85%, *p* = 0.402	Unfavorable vs. favorable BC: 42% vs. 18%, *p* = 0.028	Infectious complications: anastomotic leak, pneumonia, thoraco-abdominal abscess, subcutaneous abscess, catheter-related infection (44% vs. 61%, *p* = 0.155).	Not reported	None	Unfavorable composite body composition status, integrating muscle quantity, muscle quality, and body fat, independently predicted severe postoperative complications.
Akmercan et al. (15)	SMD < 41 HU (BMI < 25) or <33 HU (BMI ≥ 25)	Not reported separately	18% vs. 10.2%, *p* = 0.09	Perioperative mortality: 5% vs 1%, *p* = 0.08	Longer LOS in low SMD group: 6 vs. 5 day[26]s, *p* = 0.001	Muscle quantity: SMI	Muscle quality was associated with longer hospital stay and reduced overall survival; severe complications were numerically higher but not statistically significant.
Ding et al. (16)	SMD < 38.5 HU (males), < 28.6 HU (females)	OR 2.86 (*p* = 0.001)	OR 4.81 (*p* = 0.008)	Unplanned ICU transfer: 8.9% vs 0.8%, *p* = 0.003; 30-day readmission: 12.2% vs 2.4%, *p* = 0.003; Gastrointestinal complications: 10.6% vs 2.4%, *p* = 0.010	Not significant	Muscle quantity: SMI	Muscle quality was a stronger predictor of overall and severe complications compared to muscle quantity
Zhao et al. (26)	Analyzed as continuous variable	No association	Not analyzed	Not analyzed	Not analyzed	Muscle quantity: SMI, ΔSMI Adipose: SAI, VAI	Muscle quantity but not muscle quality was predictor of any complications
Zhong et al. (19)	Low SMG defined as < 1713 (males), < 1,318 (females); SMRA: median 45 HU	SMRA: OR 0.87 (*p* = 0.001); SMG: OR 0.98 (*p* = 0.001)	11.1% in L-SMG vs. 2.1% in H-SMG (*p* < 0.001)	Not analyzed	Not analyzed	Not analyzed	The novel combined parameter SMG (SMI × SMRA), integrating both muscle quantity and quality, independently predicted postoperative complications (OR 0.98) and outperformed SMI and SMRA alone (AUC: SMG 0.778 vs. SMRA 0.740 vs. SMI 0.651). Low SMG was associated with higher complication rates, major complications, and worse survival, demonstrating that combined assessment of muscle quantity and quality provides superior prognostic value than either measure alone.
Park et al. (30)	SMD: ≤ 52.4 cm^2^/m^2^ (men), ≤ 38.5 cm^2^/m^2^ (women) (weight-adapted)	Not reported	HR 1.906 (*p* = 0.032)	Postoperative 30-day mortality: Low MA (4.0% vs. 0.0%); (*p* = 0.048)	Not reported	Muscle quantity: SMI	Muscle quality nut quantity predicted major complications and 30-day mortality
Cameron et al. (27)	Analyzed as continuous variable	Not reported	Not reported	Pleural effusion: MRA (27.2 vs. 31.0 HU, *p* < 0.05)	LOS > 14 days: MRA (26.3 vs. 31.6 HU, *p* < 0.005)	Muscle quantity: SMI	Low muscle quality was associated with prolonged LOS and pleural effusion; low muscle quantity was associated with impaired pulmonary function
Murnane et al. (31)	Myosteatosis: < 41 HU (BMI < 25) or < 33 HU (BMI ≥ 25)	OR 3.03 (*p* = 0.009) (multivariate)	OR 4.33 (*p* = 0.020) (multivariate)	Anastomotic leak: 14.8% vs. 2.1% (*p* = 0.041) Pneumonia: 21.3% vs. 21.3% (*p* = 1.000) Wound infection: 13.1% vs. 2.1%, (*p* = 0.074)	15 vs. 12.5 days, (*p* = 0.058)	Muscle quantity: SMI; Adipose: SAT, VAT, IMAT	Muscle quality but not quantity predicted overall and severe complications
Huang et al. (20)	Low SMD: < 38.5 HU (males), < 28.6 HU (females)	OR 1.434 (*p* = 0.053) on univariate; Not significant on multivariate	Not significant	Not analyzed	Not reported	Muscle quantity: SMI; Adipose: SFA, VFA, VSR; Function: grip strength, gait speed	Low grip strength and age ≥80 years but not muscle quality or quantity predicted postoperative complications.
Kemper et al. (28)	Analyzed as continuous variable	Not analyzed	Not reported	Pneumonia, esophagocenteric leak, conduit necrosis, pleural effusion, pleural empyema, pulmonary embolism, respiratory failure, sepsis, acute renal failure, DVT, wound infection, intra-abdominal abscess, mediastinitis (no significant associations)	No significant association	Muscle quantity: SMI	Muscle quality was not associated with complications or LOS; muscle quantity was associated with pleural effusion, pleural empyema, and pulmonary embolism
Matsui et al. (17)	IMAC: (male: > − 0.430; female: > − 0.310)	Not reported	OR 2.260 (*p* = 0.010)	Anastomotic leakage: high vs. low IMAC 6.4% vs. 3.1% (*p* = 0.034) Infection: high vs. low IMAC: 17.3% vs. 11.7% (*p* = 0.024)	Not reported	Muscle quantity: SMI Adipose: VFA	Poor muscle quality, not muscle quantity, was associated with severe complications
Uchida et al. (18)	IMAC: male > − 0.43; female > − 0.31 (75th percentile)	Not reported	Not reported	Pneumonia: associated with high IMAC (*p* = 0.003) Anastomotic leakage: associated with high IMAC (*p* = 0.005) Infection: OR 2.391 (*p* = 0.011)	Not reported	Muscle quantity: SMI	Poor muscle quality, not muscle quantity, predicted infectious complications
Srpcic et al. (29)	MA < 30.9 HU (men), < 24.8 HU (women)	Any complication (CD ≥ II): No significant association	Not reported	Conduit complications: OR 0.238 (*p* = 0.005); Respiratory failure and in-hospital mortality: No significant association	Not reported	Muscle quantity: SMI	Muscle quality and quantity were not associated were not with perioperative complications or in-hospital mortality.
Zhuang et al. (21)	Sex-specific: males < 38.5 HU (males), < 28.6 HU (females)	21.6% vs. 14.9%, *p* = 0.007	OR 3.522 (95% CI 1.944–6.380) <0.001	Grade III: 6.2% vs. 1.5%; Grade IV: 3.3% vs. 0.7%; Grade V: 1.4% vs. 0.7%	Not reported	Muscle quantity: SMI	Muscle quality was stronger predictor of severe postoperative complications compared to muscle quantity
Lin et al. (22)	HUAC: male <52.08; female <47.75 HU GS SMG: male <2175.2; female <1493.3 HU)	Any complication (CD ≥ II): HUAC: OR 1.613 (*p* = 0.021) SMG: OR 2.247 (*p* < 0.001) SMG + GS: aOR 2.101 (*p* = 0.001)	Not reported	Not reported	Not reported	Muscle quantity: SMI Muscle function: GS Combined: SMG	Combined muscle quality and function were the strongest predictors of postoperative complications; SMG alone outperformed SMI or HUAC alone
Zhang et al. (23)	MA: male: <44.4 HU; female: <39.3 HU	Any complication (CD ≥ II): OR 12.7 (*p* = 0.017)	Not reported	Not reported	Not reported	Muscle quantity: SMI Adipose distribution: VSR	Both muscle quality and muscle quantity predicted overall complications (CD ≥ II); VSR only predicted inflammatory complications, not overall complications
Mixed cohorts							
Goda et al. (32)	MD: < 31.4 HU	CD ≥ II: OR 8.764 (*p* = 0.0225)	Not reported	Not reported	Not reported	Muscle quantity: SMI Adipose: VAI, SAI	Low muscle quality, not muscle quantity, VAI, or SAI, predicted postoperative morbidity (CD ≥ II)
Cereda et al. (33)	MA: ≤26 HU	Not analyzed	OR 4.234 (*p* = 0.006)	Not analyzed	Not reported	Muscle quantity: SMA	Both low muscle quantity and low muscle quality independently predicted major complications
Gagnat et al. (34)	Analyzed as continuous variable	OR 1.298 (*p* = 0.027)	Not reported	Not reported	Not reported	Muscle quantity: SMI Muscle strength: HGT, GST Adipose: SAT, VAT	Muscle quality, but not muscle quantity or strength, predicted surgical complications
Carvalho et al. (35)	SMD: BMI < 25: <41 HU; BMI ≥ 25: <33 HU	CD ≥ II: SMD: OR 7.82 (*p* = 0.015) HGS + SMI/SMD: OR 5.74 (*p* = 0.022)	OR 5.62 (*p* = 0.046)	Not reported	Not reported	Muscle quantity: SMI Adipose: VAT Muscle function: HGS	Muscle quality was the only independent predictor of major complications; for CD ≥ II, VAT and HGS were also predictors

CD, Clavien–Dindo classification; BMI, body mass index; BC, body composition; CI, confidence interval; OR, odds ratio; HR, hazard ratio; LOS, length of stay; ICU, intensive care unit; SMI, skeletal muscle index; SMA, skeletal muscle area; SMD, skeletal muscle density; SMRA, skeletal muscle radiation attenuation; MRA, muscle radiation attenuation; MA, muscle attenuation; MD, muscle density; HUAC, Hounsfield unit average calculation; SMG, skeletal muscle gauge; IMAC, intramuscular adipose tissue content; IMAT, intermuscular adipose tissue; VAT, visceral adipose tissue; SAT, subcutaneous adipose tissue; VATI, visceral adipose tissue index; SATI, subcutaneous adipose tissue index; SAI, subcutaneous adipose tissue index; VAI, visceral adipose tissue index; SFA, subcutaneous fat area; VFA, visceral fat area; VSR, visceral-to-subcutaneous fat ratio; HGS, handgrip strength; GS, grip strength; GST, gait speed test.

### 3.2 Methodological approaches to CT-derived myosteatosis assessment

Considerable heterogeneity was observed in CT acquisition protocols, particularly regarding contrast administration (Table 1). However, all studies assessed body composition using opportunistic preoperative CT imaging obtained as part of routine oncologic evaluation before elective surgery. Unenhanced CT scans were used in five studies (25, 27, 29, 32, 33), whereas seven studies used contrast-enhanced imaging, most commonly acquired during the portal venous phase (14, 16, 24, 28, 31) Park et al. and Uchida et al. also used contrast-enhanced CT but did not specify the acquisition phase (18, 30). In contrast, ten studies did not clearly report CT contrast status.

Anatomical landmarks and analyzed muscle groups also varied across studies (Table 1). The L3 vertebral level was used in the majority of studies. Less frequently used anatomical landmarks included combined T4 and L3 assessment (27, 32), the umbilical level (17), and the pectoralis muscle above the aortic arch (23). Most studies evaluated total skeletal muscle area, although some focused specifically on the multifidus muscles (17, 18, 34), psoas muscles (25), posterior paraspinal muscles (28), or pectoralis muscles (23).

After image selection, a wide range of segmentation software and clinical imaging platforms was used for body composition analysis, including dedicated body composition software, PACS-based systems, and other specialized imaging platforms (Table 1). Despite variability in segmentation workflows and software platforms, tissue segmentation thresholds were largely consistent across studies, with skeletal muscle typically defined within the range of −29 to +150 HU and adipose tissue between −190 to −30 HU.

In most studies, myosteatosis was assessed using attenuation-based metrics reflecting skeletal muscle radiodensity expressed in Hounsfield units. Although reported under different terminology, including skeletal muscle density (SMD) (15, 20, 21, 24, 26, 30, 31), skeletal muscle radiodensity (SMR) (14, 16, 35), skeletal muscle radiation attenuation (SMRA) (19), muscle radiation attenuation (MRA) (27, 28), muscle attenuation (MA) or mean muscle attenuation (MMA) (23, 25, 29, 32), and skeletal muscle attenuation (SMA) (33), these measures are conceptually equivalent and all represent mean CT attenuation of the analyzed muscle compartment. Additional approaches more specifically quantified fat infiltration within or around skeletal muscle, including intramuscular adipose tissue content (IMAC) (17, 18) and intermuscular adipose tissue (IMAT) (34). Some studies additionally evaluated composite indices integrating muscle quality and quantity, most notably skeletal muscle gauge (SMG) (19, 22).

Multiple approaches were used to define and analyze myosteatosis across studies. Attenuation-based metrics were analyzed either as continuous variables (26–28, 34) or using predefined cut-offs. The most common cut-offs were sex-specific, particularly <38.5 HU in males and <28.6 HU in females (14, 16, 20, 21), although alternative cut-off values were reported by Tankel et al., Srpcic et al., and Zhang et al. (23, 24, 29). Several studies used BMI-adjusted definitions, classifying myosteatosis as <41 HU in patients with BMI < 25 kg/m^2^ and <33 HU in those with BMI ≥ 25 kg/m^2^ (15, 31, 35). Other studies applied cohort-specific cut-offs, including MA ≤ 26 HU (33), MD < 31.4 HU (32), median-based SMRA and SMG categorization (19), and HUAC/SMG cutoffs (22). Matsui et al. and Uchida et al. used IMAC with sex-specific cut-offs of >−0.43 in men and >−0.31 in women (17, 18).

### 3.3 Association between myosteatosis and major postoperative complications

Associations between myosteatosis and major postoperative complications (defined as CD ≥ III), were reported in several included studies, with effect sizes generally ranging from approximately 2 to 5 (16, 17, 21, 30, 31, 33, 35). Additional studies demonstrated significantly higher rates of severe complications in patients with persistent or newly developed myosteatosis during neoadjuvant chemotherapy (24), unfavorable composite body composition scores integrating muscle quantity, muscle quality, and fat content (25), and low SMG (19). In contrast, Huang et al. found no significant association between muscle quality and major postoperative complications, whereas Akmercan et al. observed only a nonsignificant numerical increase in severe complications among patients with low SMD (15, 20).

#### 3.3.1 Association between myosteatosis and overall postoperative complications

Associations between impaired muscle quality and overall postoperative complications (CD I–V) were less consistent than those observed for major complications. Several studies reported that myosteatosis or low SMD was associated with higher overall complication rates (19, 21, 25, 31). Reported effect sizes varied considerably, ranging from modest associations to strong predictive effects exceeding OR 10 in some cohorts (14, 16, 19, 23). Similar findings were also reported in mixed surgical cohorts that included patients undergoing esophagogastric oncologic surgery (32, 33, 35). Lin et al. demonstrated that SMG was associated with postoperative complications and showed stronger predictive performance than HUAC alone or when combined with grip strength (22). Similarly, Zhong et al. found that SMG outperformed SMRA alone in predicting any postoperative complications (19).

However, not all studies confirmed these associations. Zhao et al. found no association when muscle quality was analyzed as a continuous variable (26), while Huang et al. reported only a borderline univariate association that was not significant after multivariable adjustment (20). Similarly, Srpcic et al. found no association between muscle quality and postoperative complications defined as CD ≥ II (29).

Impaired muscle quality was associated with a broad range of postoperative complications, particularly infectious, pulmonary, and conduit-related complications. Reported infectious complications included anastomotic leakage, wound infection, thoraco-abdominal abscess, conduit complications, subcutaneous abscess, catheter-related infection, mediastinitis, sepsis, and general postoperative infections (17, 18, 25, 29, 31). Pulmonary complications such as pneumonia, pleural effusion, pleural empyema, respiratory failure, and pulmonary embolism were also commonly evaluated, with some cohorts demonstrating significant associations with impaired muscle quality (18, 27), whereas others found no significant relationship (28, 29). Myostatosis was also associated to unplanned ICU admission, 30-day readmission, gastrointestinal complications, conduit complications, and 30-day mortality (14–16, 29, 30). However, findings for individual complications were not entirely consistent across cohorts.

#### 3.3.2 Length of stay

Associations between impaired muscle quality and prolonged length of stay (LOS) were reported in several studies. Akmercan et al. found significantly longer hospital stay in patients with low SMD (15), while Cameron et al. reported lower muscle radiodensity in patients with LOS > 14 days (27). Murnane et al. also observed longer hospitalization in patients with myosteatosis, although the difference did not reach statistical significance (31). In contrast, Tankel et al., Kemper et al., and Srpcic et al. found no significant association between muscle quality and LOS (24, 28, 29). Overall, impaired muscle quality appears to be associated with prolonged hospitalization in some esophagogastric surgery cohorts, although the evidence remains heterogeneous.

#### 3.3.3 Comparison to other body composition variables

Myosteatosis was usually evaluated alongside other body composition measures, most commonly muscle quantity, adiposity indices, and, in some studies, functional measures such as grip strength or gait speed (Table 2). Across many cohorts, myosteatosis appeared to be more consistently associated with postoperative morbidity than muscle quantity alone, particularly in studies where SMI was weaker or non-significant while SMD, MA, IMAC, or IMAT remained predictive (16–18, 21, 24, 31, 32, 35). In some studies, however, both muscle quality and muscle quantity predicted postoperative outcomes, without clear evidence that one measure consistently outperformed the other (23, 33). As discussed previously, some studies reported improved prognostic performance of composite parameters integrating muscle quality with muscle quantity, adiposity, or physical function, including SMG and composite body composition scores (19, 22, 25). However, findings were not entirely consistent across studies, with some cohorts reporting stronger associations for functional measures or muscle quantity than for muscle quality alone (20, 26).

## 4 Discussion

In this systematic review, CT-derived myosteatosis was frequently associated with adverse postoperative outcomes in patients undergoing esophagogastric oncologic surgery, particularly major postoperative complications. Across many cohorts, impaired muscle quality demonstrated stronger and more consistent associations with postoperative morbidity than muscle quantity alone, although findings for overall complications, specific postoperative events, and length of stay were less consistent.

### 4.1 Methodological approaches to CT-derived myosteatosis assessment

Considerable heterogeneity in CT acquisition protocols represents an important methodological limitation of the available literature. Intravenous contrast administration and acquisition phase may influence skeletal muscle attenuation measurements and potentially affect myosteatosis classification and comparability across studies (36). Although correction factors have been proposed to harmonize attenuation measurements across different CT phases (37), inconsistent reporting of contrast status remains a major limitation of current body composition research. Nevertheless, opportunistic preoperative CT imaging represents a major practical advantage, enabling assessment of myosteatosis without additional imaging, radiation exposure, or cost.

Variation in anatomical level and muscle selection is clinically relevant because CT-derived muscle measures are not fully interchangeable across regions. The L3 level is widely used because single-slice skeletal muscle area at this level correlates strongly with whole-body skeletal muscle volume and is therefore commonly treated as the reference landmark in cancer body composition research (38). However, thoracic landmarks, pectoralis muscles, psoas-only measures, and paraspinal or multifidus assessments may capture different regional muscle compartments and may not provide equivalent estimates of global muscle status (39, 40). This variability limits direct comparison between studies and supports the need for standardized anatomical landmarks or validated conversion approaches when L3 imaging is unavailable.

Despite substantial heterogeneity in terminology, most studies assessed myosteatosis using conceptually similar attenuation-based CT metrics reflecting skeletal muscle radiodensity. Although the diverse nomenclature may suggest that multiple distinct parameters were evaluated, most attenuation-based measures represent the same underlying construct, namely mean CT attenuation of skeletal muscle as a surrogate of fatty infiltration and muscle quality. In contrast, approaches such as IMAC and IMAT evaluate more specific adipose tissue compartments and are therefore not directly interchangeable with standard attenuation-based metrics. Composite indices such as skeletal muscle gauge further integrate muscle quality and quantity into a single parameter. Overall, these findings highlight persistent methodological variability and the need for more standardized terminology in CT-derived myosteatosis research (36).

The variability in myosteatosis cut-offs observed in the present review is consistent with findings from other gastrointestinal cancer populations. In colorectal cancer, Han et al. reported heterogeneity in measurement methods and diagnostic criteria and concluded that future studies should clarify CT acquisition protocols and diagnostic criteria (11). Similarly, studies included in the pancreatic cancer meta-analysis by Chen et al. used variable diagnostic approaches (12). Thus, the cut-off variability observed in esophagogastric surgery is not unique, but reflects a broader limitation of CT-derived myosteatosis research across gastrointestinal oncology.

### 4.2 Association between myosteatosis and postoperative complications

The association between myosteatosis and major postoperative complications observed in the present review is consistent with findings across other oncologic surgical populations. In colorectal cancer, Han et al. reported low-to-moderate certainty evidence linking myosteatosis with severe postoperative complications, despite substantial heterogeneity in imaging methodology and diagnostic definitions (11). Similarly, Chen et al. demonstrated in a meta-analysis of pancreatic cancer that myosteatosis was associated with postoperative complications and adverse survival outcomes despite variability in assessment approaches (12). Comparable findings were reported in urologic, gynecologic, and thoracic oncology cohorts, where impaired muscle quality predicted major complications after radical cystectomy (41), ovarian cancer surgery (42), and thoracoscopic lobectomy for non-small cell lung cancer (43).

The less consistent association between myosteatosis and overall postoperative morbidity observed in the present review is in line with broader oncologic surgery literature, where myosteatosis appears to predict severe complications more reliably than heterogeneous composite endpoints such as any complication. In colorectal surgery, several studies reported associations between impaired muscle quality and overall morbidity (44), whereas others found no significant association (45). Similarly, in gynecologic oncology, impaired muscle quality was associated with any 30-day complication in ovarian cancer cohorts, but available evidence remains less standardized than for major complications (42). This inconsistency likely reflects differences in outcome definitions, because CD I–V combines minor deviations from recovery with clinically severe events, as well as differences in CT methodology, cut-offs, and whether muscle quality was analyzed alone or within composite indices such as SMG.

The association between impaired muscle quality and specific postoperative complications observed in the present review is also consistent with findings from other oncologic surgical populations. In colorectal surgery, myostatosis has been associated with anastomotic leakage, postoperative pneumonia, respiratory and cardiac complications, prolonged recovery, and readmission (46, 47). Similar findings have been reported in gynecologic oncology, where impaired muscle quality predicted postoperative infections, functional decline, and discontinuation of adjuvant chemotherapy after ovarian cancer surgery (48), as well as in thoracic oncology, where low muscle quality was associated with major postoperative morbidity following lung cancer resection (43). Collectively, these findings suggest that CT-derived myosteatosis may reflect systemic vulnerability to infectious, pulmonary, and recovery-related complications across multiple oncologic surgical populations, although associations with individual complications remain less consistent than those observed for major postoperative morbidity.

The inconsistent association between impaired muscle quality and LOS in esophagogastric surgery is also reflected in other oncologic cohorts. In colorectal surgery, several studies reported associations between low muscle quality and longer hospitalization or delayed recovery (10, 49), whereas others found no significant association (47, 50) In gynecologic and orthopedic oncology cohorts, lower muscle quality was also associated with longer LOS or discharge-related functional decline (51, 52). Overall, LOS appears to be a less specific endpoint than major complications, as it is influenced not only by postoperative morbidity but also by institutional discharge practices, rehabilitation pathways, social factors, and perioperative care protocols.

Muscle quantity is already an established body composition marker in esophagogastric cancer surgery, with meta-analyses showing that low SMI-defined sarcopenia is associated with postoperative complications and poorer survival after esophagectomy and gastrectomy (9). Similar associations between low muscle quantity and adverse postoperative outcomes have also been reported across colorectal, pancreatic, gynecologic, thoracic, and urologic oncology surgery populations (53–57). However, the present findings suggest that CT-derived myosteatosis may provide complementary, and in some cohorts stronger, prognostic information than muscle quantity alone. Several included studies found that myosteatosis remained associated with postoperative morbidity when sarcopenia was weaker or non-significant (16–18, 31, 32, 35), whereas other studies reported that both muscle quality and quantity contributed to risk (14, 23). This pattern is consistent with the broader shift from assessing muscle mass alone toward evaluating muscle composition and function. Composite approaches such as SMG and body composition scores may therefore be particularly useful because they integrate muscle quantity, quality, adiposity, or function into a more global measure of physiological reserve (19, 25). The albumin–myosteatosis gauge (AMG), which combines skeletal muscle radiodensity with serum albumin, has been proposed as another composite biomarker integrating muscle quality with nutritional and inflammatory status. Although currently evaluated primarily in pancreatic cancer, AMG represents a promising direction for future studies of perioperative risk stratification (58). Nevertheless, inconsistent findings across studies indicate that muscle quality should not yet be viewed as replacing muscle quantity, but rather as a complementary marker that may improve perioperative risk stratification when assessed alongside SMI and functional measures.

Several important limitations of the current literature should be acknowledged. Considerable methodological variability persists across studies regarding CT acquisition protocols, contrast administration, anatomical landmarks, analyzed muscle groups, segmentation software, terminology, and diagnostic cut-offs used to define myosteatosis. In particular, inconsistent reporting of CT contrast phase represents a major challenge, as intravenous contrast significantly influences skeletal muscle attenuation values and may affect the classification of myosteatosis. Similarly, the absence of universally accepted diagnostic thresholds limits comparability between studies and complicates translation into clinical practice. Future research should therefore prioritize greater standardization of CT-derived muscle quality assessment, including harmonized acquisition protocols, consistent reporting of contrast phase, and consensus-based diagnostic cut-offs. At the same time, routine implementation of CT-based body composition analysis remains limited by the need for specialized software, image processing, and radiologic expertise. The development of inexpensive, automated, and externally validated artificial intelligence–based segmentation algorithms may substantially improve scalability and facilitate integration into routine perioperative workflows. Finally, although our search identified one additional potentially relevant study, the full text could not be obtained despite attempts through institutional access and direct correspondence with the authors. Consequently, the study could not be assessed for eligibility and was not included in the review. In addition, although this systematic review was conducted and reported in accordance with the PRISMA 2020 guidelines, it was not prospectively registered in PROSPERO.

Future research should extend beyond CT-derived measures and investigate complementary approaches to muscle quality assessment, including ultrasound-derived measures, functional testing, and multimodal assessment strategies integrating muscle quantity, quality, strength, and physical performance. Finally, most currently available evidence remains retrospective and observational, highlighting the need for prospective multicenter studies with standardized methodology to better define the clinical utility of myosteatosis in perioperative risk stratification.

## 5 Conclusion

CT-derived myosteatosis appears to be consistently associated with major postoperative complications in patients undergoing esophagogastric oncologic surgery and, in several cohorts, demonstrated stronger prognostic associations than muscle quantity alone. Associations with overall morbidity, specific postoperative complications, and length of stay were more heterogeneous, likely reflecting variability in outcome definitions, imaging methodology, and diagnostic thresholds. Nevertheless, the available evidence suggests that impaired muscle quality represents an important marker of reduced physiological reserve and perioperative vulnerability. Composite approaches integrating muscle quality with muscle quantity, adiposity, or functional measures may further improve risk stratification compared with isolated body composition parameters. Future research should prioritize methodological standardization, prospective multicenter validation, and development of automated and clinically accessible assessment tools to facilitate integration of muscle quality evaluation into routine perioperative care.

## Data Availability

The original contributions presented in the study are included in the article/Supplementary material, further inquiries can be directed to the corresponding author.
