# Supplementary material for: CT-derived myosteatosis as a predictor of postoperative outcomes in esophagogastric cancer surgery: a systematic review
Source: Front Med (Lausanne). 2026 Jul 21;13:1885900. doi: 10.3389/fmed.2026.1885900 (PMC13433744; doi:10.3389/fmed.2026.1885900)
Supplement: Supplementary file 1 [file Table_1.DOCX]

**Supplementary File 1. Database-specific search strategies**

**PubMed/MEDLINE**

Search date: June 20, 2026

(("myosteatosis"[Title/Abstract])
OR ("muscle quality"[Title/Abstract])
OR ("skeletal muscle density"[Title/Abstract])
OR ("skeletal muscle radiodensity"[Title/Abstract])
OR ("muscle attenuation"[Title/Abstract])
OR ("muscle radiodensity"[Title/Abstract])
OR ("intramuscular adipose tissue"[Title/Abstract])
OR ("intermuscular adipose tissue"[Title/Abstract])
OR ("IMAT"[Title/Abstract])
OR ("IMAC"[Title/Abstract])
OR ("skeletal muscle gauge"[Title/Abstract]))

AND

(("Stomach Neoplasms"[Mesh])
OR ("Esophageal Neoplasms"[Mesh])
OR ("gastric cancer"[Title/Abstract])
OR ("stomach cancer"[Title/Abstract])
OR ("gastric carcinoma"[Title/Abstract])
OR ("esophageal cancer"[Title/Abstract])
OR ("oesophageal cancer"[Title/Abstract])
OR ("esophageal carcinoma"[Title/Abstract])
OR ("oesophageal carcinoma"[Title/Abstract])
OR ("esophagogastric cancer"[Title/Abstract])
OR ("oesophagogastric cancer"[Title/Abstract])
OR ("gastroesophageal junction cancer"[Title/Abstract]))

AND

(("Surgical Procedures, Operative"[Mesh])
OR ("Postoperative Complications"[Mesh])
OR gastrectomy[Title/Abstract]
OR esophagectomy[Title/Abstract]
OR surgery[Title/Abstract]
OR surgical[Title/Abstract]
OR postoperative[Title/Abstract]
OR perioperative[Title/Abstract]
OR complication*[Title/Abstract]
OR outcome*[Title/Abstract])

**Embase**

Search date: June 20, 2026

('myosteatosis' OR 'muscle quality' OR 'skeletal muscle density' OR 'skeletal muscle radiodensity' OR 'muscle attenuation' OR 'muscle radiodensity' OR 'intramuscular adipose tissue' OR 'intermuscular adipose tissue' OR IMAT OR IMAC OR 'skeletal muscle gauge')

AND

('gastric cancer' OR 'stomach cancer' OR 'gastric carcinoma' OR 'esophageal cancer' OR 'oesophageal cancer' OR 'esophageal carcinoma' OR 'oesophageal carcinoma' OR 'esophagogastric cancer' OR 'oesophagogastric cancer' OR 'gastroesophageal junction cancer')

AND

(gastrectomy OR esophagectomy OR surgery OR postoperative OR perioperative OR complication* OR outcome*)

**Web of Science Core Collection**

Search date: June 20, 2026

TS=("myosteatosis" OR "muscle quality" OR "skeletal muscle density" OR "skeletal muscle radiodensity" OR "muscle attenuation" OR "muscle radiodensity" OR "intramuscular adipose tissue" OR "intermuscular adipose tissue" OR IMAT OR IMAC OR "skeletal muscle gauge")

AND

TS=("gastric cancer" OR "stomach cancer" OR "gastric carcinoma" OR "esophageal cancer" OR "oesophageal cancer" OR "esophageal carcinoma" OR "oesophageal carcinoma" OR "esophagogastric cancer" OR "oesophagogastric cancer" OR "gastroesophageal junction cancer")

AND

TS=(gastrectomy OR esophagectomy OR surgery OR postoperative OR perioperative OR complication* OR outcome*)

**Scopus**

Search date: June 20, 2026

TITLE-ABS-KEY("myosteatosis" OR "muscle quality" OR "skeletal muscle density" OR "skeletal muscle radiodensity" OR "muscle attenuation" OR "muscle radiodensity" OR "intramuscular adipose tissue" OR "intermuscular adipose tissue" OR IMAT OR IMAC OR "skeletal muscle gauge")

AND

TITLE-ABS-KEY("gastric cancer" OR "stomach cancer" OR "gastric carcinoma" OR "esophageal cancer" OR "oesophageal cancer" OR "esophageal carcinoma" OR "oesophageal carcinoma" OR "esophagogastric cancer" OR "oesophagogastric cancer" OR "gastroesophageal junction cancer")

AND

TITLE-ABS-KEY(gastrectomy OR esophagectomy OR surgery OR postoperative OR perioperative OR complication* OR outcome*)

**Cochrane Library**

Search date: June 20, 2026

("myosteatosis" OR "muscle quality" OR "skeletal muscle density" OR "skeletal muscle radiodensity" OR "muscle attenuation" OR "muscle radiodensity" OR "intramuscular adipose tissue" OR "intermuscular adipose tissue" OR IMAT OR IMAC OR "skeletal muscle gauge")

AND

("gastric cancer" OR "stomach cancer" OR "gastric carcinoma" OR "esophageal cancer" OR "oesophageal cancer" OR "esophageal carcinoma" OR "oesophageal carcinoma" OR "esophagogastric cancer" OR "oesophagogastric cancer" OR "gastroesophageal junction cancer")

AND

(gastrectomy OR esophagectomy OR surgery OR postoperative OR perioperative OR complication* OR outcome*)

In addition to database searching, the reference lists of all eligible studies and relevant review articles were manually screened to identify further potentially eligible publications. No language restrictions were applied during the search process.
